# Development and evaluation of the Rural and Northern Community Focused Model of COPD Care (RaNCoM)

**DOI:** 10.1186/s12890-023-02683-2

**Published:** 2023-10-20

**Authors:** Shannon Freeman, Laura Peach, Christopher Ross, Kathy Marchal, Anthon Meyer, Kelly Skinner

**Affiliations:** 1 School of Nursing, University of Northern British Columbia, British Columbia, 3333 University Way, Prince George, V2N 4Z9 Canada; 2 Centre for Technology Adoption for Aging in the North, University of Northern British Columbia, Prince George, British Columbia, Canada; 3 School of Public Health Sciences, University of Waterloo, Waterloo, ON Canada; 4 Fort St, James Health Centre, Fort St. James, British Columbia, Canada; 5 Rural Coordination Centre of BC, British Columbia, Canada

**Keywords:** Chronic obstructive pulmonary disease, Primary care, Rural, Northern, Model, Process evaluation

## Abstract

**Background:**

The prevalence of COPD continues to rise. To address the challenges to provide high quality COPD care in rural and northern communities, leaders in one rural and northern community in Western Canada sought to change the culture of COPD screening and care. Recognizing effective assessment, diagnosis, and treatment for patients with COPD are crucial to improve outcomes, a program was developed between 2012 and 2021 to enhance primary care for COPD patients.

**Methods:**

A process evaluation was undertaken to assess program development, implementation, mechanisms of impact, and context of COPD program. Qualitative thematic analysis of stakeholder interviews (*n* = 11) and a document review (*n* = 60; ~ 500 pages) of key clinic documents was conducted.

**Results:**

We describe five phases of the COPD program’s development (Survive; Reorganize and Stabilize; Assess and Respond; Build and Refine; and Sustain and Share), highlighting areas of innovation. Outreach and localizing resources improved access to the program. Acquiring secured physician compensation, capturing quality data, and improving patient and provider self-efficacy built the capacity of the system and stakeholders within it. Finally, relationships were forged through building an integrated facility, collaborative networking, and patient engagement. Key elements of program implementation included the resources (infrastructure, software, operational) required to ensure operation.

**Conclusion:**

Team-based care and service integration enhanced care capacity and the health network. Focused use of infrastructure and resources supported the people in the care system. Upholding a shared value of relationship is critical to deliver robust and sustainable rural healthcare. Quality improvement requires investment in rural community healthcare resources.

## Background

Chronic Obstructive Pulmonary Disease (COPD) is a leading cause of hospital admission and death globally [1, 2]. In North America, COPD is the third leading cause of death [3], and the prevalence of this condition has increased in Canada by 82% between 2001–2013 [4]. Moreover, COPD is prominent in many northern communities across Western Canada [5]. Estimates indicate over 17% of the Canadian adult population is affected by COPD [4], and up to one in four adults over the age of 35 will develop COPD during their lifetime [6]. It is estimated that 15% of Canadians aged 65–69 and 26% of Canadians aged 85 years and older are living with COPD. A review of Canadian studies found that COPD patients average 0–4 emergency department visits per year and average up to 5 physician visits annually posing a substantial burden to the Canadian healthcare system [7].

Healthcare systems around the world are increasing their quality of care by focusing on the processes of their existing programs [8]. To achieve this, healthcare administrators are adopting techniques and tools from health and evaluation disciplines as well as from management and business administration disciplines. Canadian healthcare settings using chronic disease management implementation often draw on quality improvement frameworks, such as the Plan-Do-Study-Act method, Six Sigma and Total Quality Management and are using business modeling techniques and simulations to better understand their processes [8–11]. Simultaneously, to better understand the dynamics and actual operations of programs, the healthcare field prioritizes evaluation methodologies, including program evaluation, to understand the strengths and weaknesses of a program and to search for explanations of the program’s successes and failures [12].

Disparities exist in COPD prevalence, severity, and care between rural and urban communities [13, 14]. The prevalence of COPD is disproportionately higher in many rural areas compared to urban areas [13]. Further, the risk for severe COPD has also been reported to be more prevalent in rural areas [15]. Recognizing that effective assessment, diagnosis, and treatment for patients with COPD are essential to change the disease trajectory and improve patient outcomes, a program was developed to provide enhanced care for COPD patients in the primary care context in a rural and northern community in a Western Canadian province [16, 17]. The objective of this study was to employ a qualitative approach to describe the new innovative model of rural COPD service delivery and the process undertaken to develop the program, as well as identify stakeholder perceptions of program outcomes and impact. The process evaluation framework described by Moore and colleagues, which investigates the implementation, mechanisms of impact, and context of an intervention, as well as the relationship among them, was selected to guide this work in a retrospective manner [18].

## Methods

### Community background

The study was conducted in a small, rural, northern community in Western Canada, approximately 2 hours by car from the nearest urban centre. The municipality where this community was located has a population less than 1,500 people [19]. The community health centre serves a wider population (approximately 5,000 persons) that includes surrounding First Nations and rural communities. Forty-three percent of the population is over the age of 45 years [19]. Further, woodburning appliances and smoking are highly prevalent. Historically, the community had difficulty retaining permanent full-time physicians to meet community healthcare needs. From the 1980s to late-2000s, for instance, only one physician was staffed at the community health centre to serve the area, with intermittent support from short locum contracts. As such, episodic acute emergency care was the norm for years without the capacity to build health programs targeting prevalent chronic diseases, such as COPD.

A snowball recruitment strategy was used to invite key stakeholders to share their knowledge of and experience with the COPD program, its history, and its perceived challenges and successes. Recruitment posters were also placed at the clinic reception desk to attract the interest of clinic patients, who were then referred to the research team by clinic staff. Semi-structured interviews were conducted by two members of the research team (LP and CR) with clinic management staff (*n* = 5) and healthcare providers (*n* = 5) involved in either the development of the COPD program or in the current provision of COPD care in the town along with one clinic patient. In early 2021, interviews were completed over Zoom, audio recorded, and transcribed verbatim, and a document review of approximately 60 documents (550 pages in length) published between 2011 and 2020 was conducted. Documents were collected by the research team from key stakeholders to provide further detail to the program’s development and implementation process, as well as verify or clarify interviewee recollections of various program stages. All included documents contained de-identified and anonymous information. No financial reporting documents nor patient records were included. The final document pool included a multitude of clinic document types such as provincial practice guidelines, meeting minutes, workflow graphic models, academic journal publications, presentation slides, and training materials.

### Analysis

Interview transcriptions were imported into NVivo qualitative coding software and thematic analysis was undertaken [20, 21]. To establish intercoder reliability, limit bias, and establish rigor, two researchers (LP and JR) each selected the same 5 transcripts randomly and coded them separately to begin an inductive-deductive analysis process [22]. This process incorporated both a pre-determined strategy (deductive) that drew from theoretical concepts to inform interpretation of the data – in this case, reference was made to Moore et al.’s framework – as well as an emergent strategy (inductive) that identified representative data to support findings and deepens understanding of themes. The researchers then met to discuss the commonalities and differences between their preliminary findings, which resulted in an overwhelming amount of agreement despite some small negotiations around the names of themes assigned independently. This process informed the development of a final single coding framework that was applied to all transcripts by one member of the research team (LP). Once this process was complete, the findings were organized into final key themes.

Two research team members (SF and CR) selected relevant documents which were then scanned and imported into NVivo 12 qualitative analysis software [21]. Ultimately, the same coding framework that was applied to the interviews (LP) was also used to conduct both a longitudinal [23] and thematic analysis of the documents’ contents, identifying (dis)continuities and pattern changes across the program’s lifespan and enriching understanding of other program themes identified in stakeholder narratives related to the processes of program development and implementation. New codes were assigned specifically for organization purposes (e.g., publication year, document type). Harmonized ethics approval was received from the ethics committee of University of Northern British Columbia and the Northern Health Authority (H20-01877). All research activities were carried out in accordance with the Tri-Council Policy Statement on Research Ethics. All participants provided informed consent prior to participation in project activities.

## Results

**Fig. 1:**
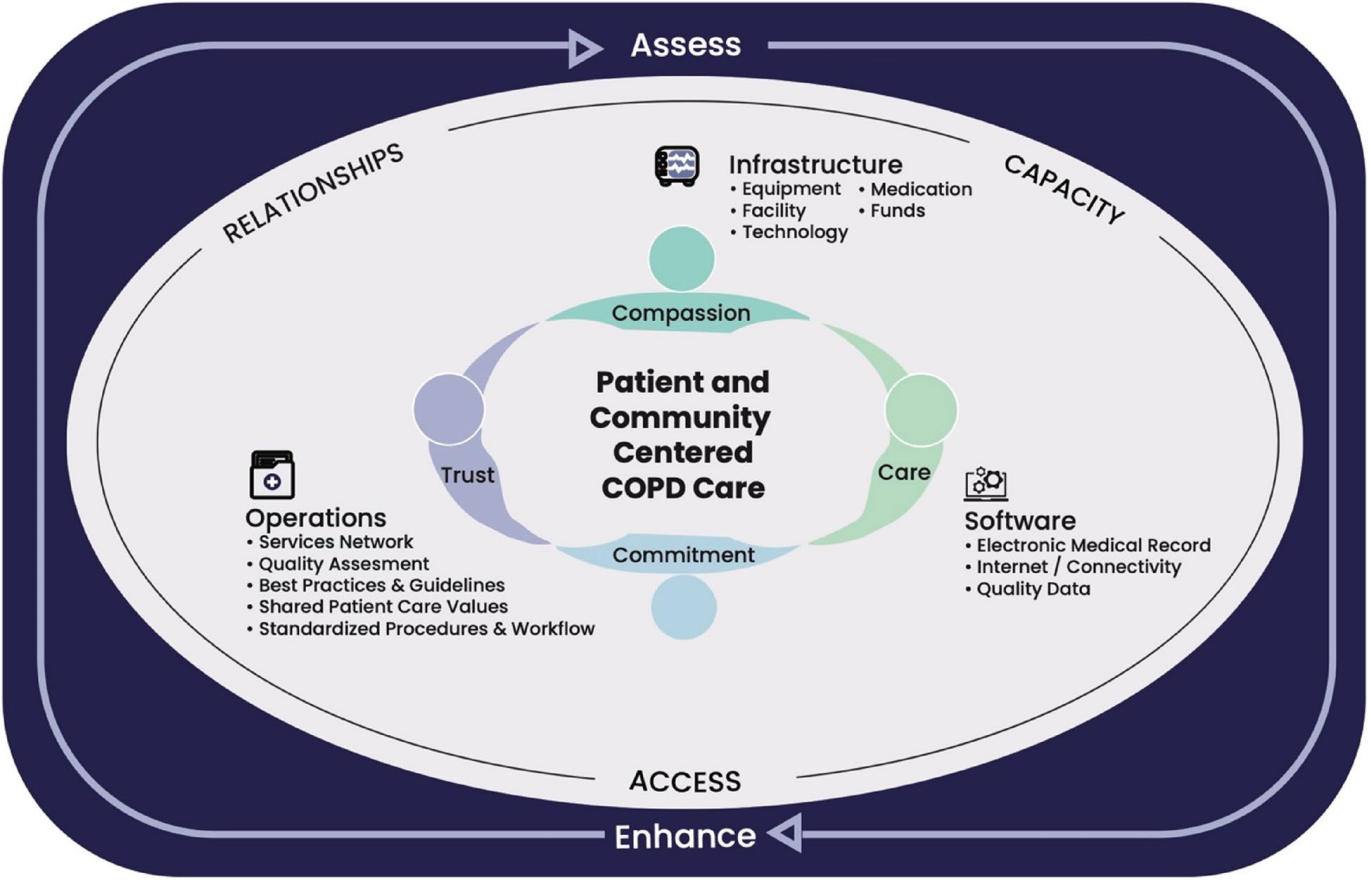
Graphic Depiction of the Components and Functioing of the Rural and Northern Community Focused Model of COPD Care (RaNCoM)

As the single coding framework was developed with consideration of Moore et al.’s framework, the key findings also aligned with this guide to process evaluations of complex interventions which incorporated three key functions of process evaluation: implementation, impact, and context [18]. As such, three main findings are reported. First, a program development timeline describes the processes and actions required to create and implement the present Rural and Northern Community Focused Model of COPD Care (RaNCoM). Second, access, capacity and relationships were identified as significant program outcomes that benefited program participants. Finally, the remaining program challenges reveal ways the program can be further improved to meet patient and community needs. Figure 1 details how RaNCoM encapsulates the areas of innovation intrinsic to the delivery of the COPD program.

### Program development

**Fig. 2:**
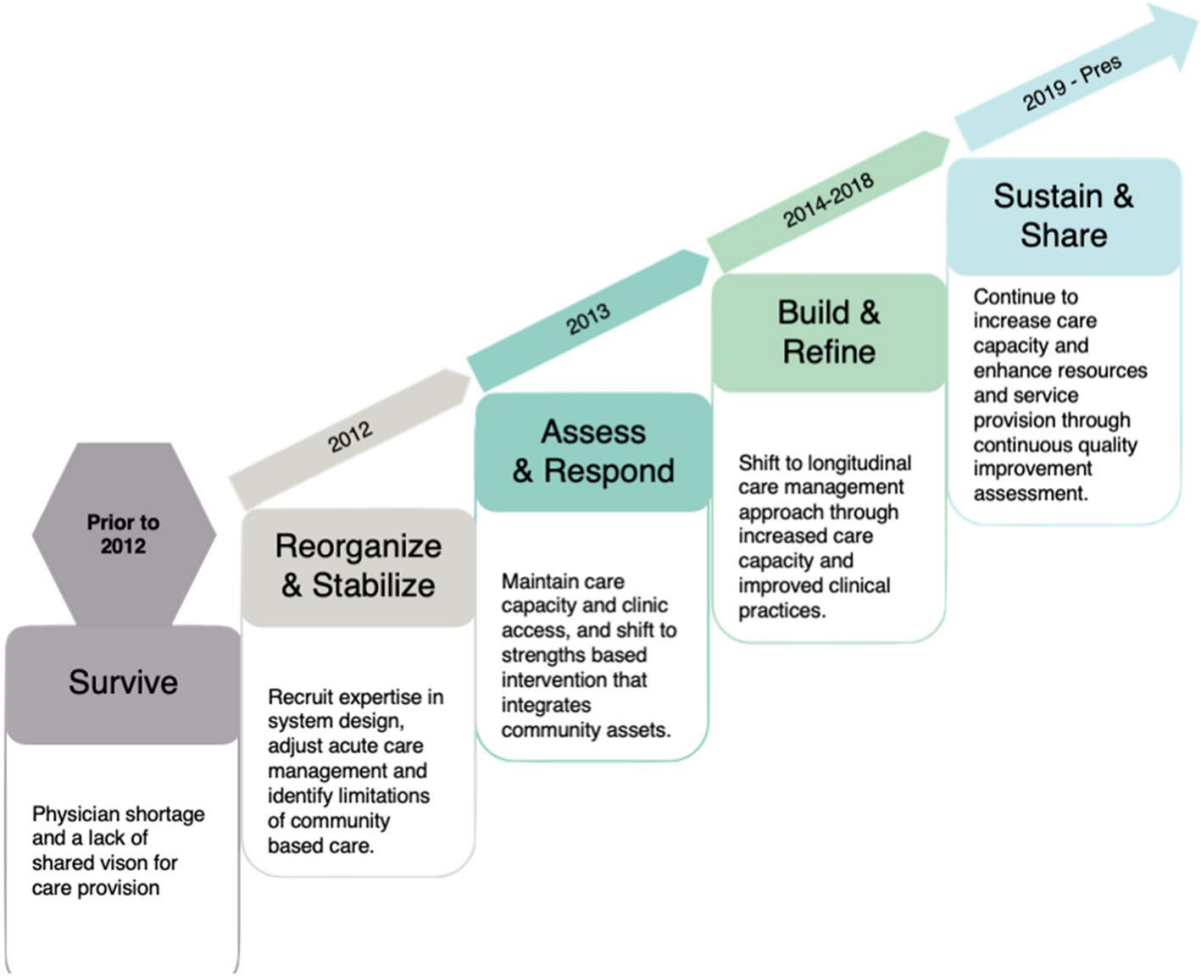
Rural and Northern Community Focused Model of COPD Care (RaNCoM) Program Development Timeline and Key Phases 2012—2021

The processes and actions critical to RaNCoM development, evolution, and implementation were detailed across five program development phases: 1) Survive, 2) Reorganize and Stabilize, 3) Assess and Respond, 4) Build and Refine, and 5) Sustain and Share (Fig. 2).

### Survive

Prior to the COPD program’s implementation in 2012, the clinic was in a phase of survival. Fluctuating physician numbers due to short-term locum contracts, staff shortages, and overall poor retention created a lack of consistent patient care and limited care provision capacity. As early as 2008, the physician-to-population ratio for the area was determined to be a minimum of six full-time physicians, which had never been secured. One care provider working at this time relayed that there was no shared vision between clinicians for approaching and providing care:

The previous care model was one of a siloed approach, [with] fragmented services [and a] revolving door mentality… where physicians provided services to an overwhelming rural, complex environment and patient panel*.* (Care Provider 3)

The fee-for-service payment model exacerbated this situation by incentivizing the quantity of patients treated by physicians rather than the type of care provided. Further, the Emergency Department (ED) at the small local hospital was the primary treatment site in this period. The community had a high prevalence of unmanaged chronic disease that led to numerous presentations of acute exacerbations and perpetual episodic care.

### Reorganize and stabilize

In 2012, the COPD program began its development in tandem with a clinic-wide reorganization phase characterized by an arrangement with the local health authority to oversee physician contracts under an Alternative Payment Plan [24]. A recruitment coordinator worked with community stakeholders to obtain additional physicians for the region. Concurrently, a not-for-profit primary care organization was established to oversee clinic management and work towards local health service integration, including development of a shared electronic medical record (EMR) linking the hospital, physicians, and First Nations resources and health centres. This integration began to control and improve the care setting by stabilizing acute care and identifying the care limitations needing attention, which included the physician shortage and retention challenges preventing adequate management of chronic diseases well as inconsistencies in EMR coding that compromised data quality. The care team also began taking stock of the community context and its needs. One care provider explained:

[As] the only two doctors on the ground, they saw a lot of COPD exacerbations coming through the Emergency Room, so that [was] the impetus for them starting proper COPD management with these patients. (Care Provider 2).

They recognized a high prevalence of COPD, among other chronic diseases, and identified it as a priority health issue that, if addressed, would lead to improvements in both community health and clinic functioning.

### Assess and respond

The process of incentivizing quality care resulted in increased physician care capacity secured under the Alternative Payment Plan by 2013. Quality improvement procedures assessing the viability of this payment model were also put in place, as confirmed by an internal clinic memo of the same year. This transformed care provision from an ‘acute care management approach’ to a ‘chronic disease management approach’ informed by EMR data and presentation trends in the local hospital ED. Under this approach, the launch of the COPD program was marked by the implementation of several core activities: EMR protocols, patient panel distribution and scheduling, professional standards and guidelines, and COPD assessment. One care provider spoke about the coding and search protocols employed in the EMR system to identify patients with, or a clinical suspicion of, COPD:

I started pulling a lot of reports through our medical electronic records system to identify at-risk patients [for early detection] …We actually pulled a few people and sent them for a spirometry test. (Care Provider 2)

Patient panels, or groups of patients assigned to physicians for care provision, were then distributed equitably among physicians to ensure patients were properly enrolled to individualized disease management plans. Walk-in appointment scheduling, a Practice Support Program method, was also implemented to enhance clinic access. Further, the clinic purposefully implemented professional standards, policies, and guidelines outlined by the regional health authority and the provincial College of Physicians and Surgeons. Progress to consistently implement the COPD Assessment Test™ to assist with and track disease management was noted [25, 26].

### Build & refine

In 2014, the clinic continued increasing its care capacity to support the care model’s shift to a chronic disease management focus. Staff included six full-time physicians and one nurse practitioner, meeting the requirement determined in 2008, as well as three locum physicians and additional medical students as extra support. A Practice Support Coach was also hired to improve clinic management, such as enhancing the Medical Office Assistant role to alleviate physician workloads and ensure complex care registries were upheld through patient bookings and recalls. One management staff recalled:

It took everybody. It took the physician to meet the patient, it took the spirometry to get the diagnosis, it took the recall to bring that patient back in and start a recall process. (Management Staff 1)

Additionally, the team identified the need for a specialist outreach program and built it over three years through soliciting specialists and utilizing telehealth and face-to-face specialist appointments at the clinic.

Continued improvements to clinical practice implementation included establishing workflow processes for physicians between the hospital and walk-in clinic environments, as well as beginning to implement the team-based care model. One management staff shared the benefits of these practice changes:

It’s that teamwork mentality of, you know, the physicians working with management and the staff to say, ‘this is what we need, and this is why we need it.’ [And I can go to the doctors] and say, ‘hey, I need this updated for my sake.’ So, we all work really well together. (Management Staff 4)

Enhancing and enforcing shared EMR documentation practices was critical to this team-based practice, which ensured patient medical records were accurate, maintained, and accessible to all staff to harmonize clinic operation and share the workload of documentation. Frequent quality improvement assessments were also implemented according to clinic workflow documents (*n* = 7) and internal memos (*n* = 3), which gathered high quality data on program efficacy through tools like patient and provider experience surveys and short, iterative PDSA cycles [11].

Combined, these clinical practice improvements initiated a longitudinal approach to continuous care, setting the foundation for sustainable, effective clinic operation to be established in the following years. In 2015 for instance, a scheduling process was developed to address the need for pre-booked and same-day appointments based on medical urgency, and patient EMRs were made accessible to allied health professionals to create wrap-around services and solidify broader localized service integration. The acquisition of in-house spirometry, and clinic staff roles (e.g., Medical Office Assistants) in 2017 were further enhanced by respiratory technologist training to support COPD screening activities, as confirmed by internal clinic emails (*n* = 5) spirometry training materials (*n* = 2). An arranged partnership with a metropolitan hospital established efficient spirometry readings, which decreased results wait-times from 9 weeks to 48 hours. Further, cultural humility and safety training for clinic staff was planned for intentional implementation to better serve clients from the surrounding First Nations communities. In 2018, a clinic expansion created space for comprehensive, multi-level care and a spirometry program. Simultaneously, a new integrated facility was planned for construction to house physicians, management, and health authority staff under one roof. This plan included enhanced outreach and integration planning for surrounding First Nation communities for shared resources and care activities, including traditional wellness provision, improved internet connection for telehealth outreach, and additional permanent physician positions. Several interviewees mentioned the important social development that occurred in this phase through building patient and community trust. Table 1 summarizes the resources, perceived mechanisms of action, and the outcomes of this program development stage. Together, these actions demonstrated to patients and the community that long-term and meaningful transformation was taking place.

**Table 1:** Inputs, mechanisms of action, and outcomes of the build and refine program development stage of the Rural and Northern Community Focused Model of Care (RaNCoM)

Inputs	Mechanisms of Action	Outcomes
**Infrastructure** ⦁ Spirometry equipment ⦁ Tele-health technology ⦁ New clinic facility ⦁ Medication ⦁ Funds	**Improved Facility** ⦁ Increased space ⦁ Provision of multi-level care	**Access** ⦁ Local spirometry access ⦁ Enhanced medication coverage and use
**Software** ⦁ Electronic Medical Record ⦁ Quality data	**Operations and Policies** ⦁ Implement standardized procedures and workflow ⦁ Frequent quality assessment cycles ⦁ Apply best practices and guidelines from health and professional authorities ⦁ Network between regional services, local integration	**Capacity** ⦁ Improved patient and physician self-efficacy ⦁ Improved patient outcomes
**Human Resources** ⦁ Adequate clinic staff ⦁ Engaged patient pool Time	**Patient Outreach** ⦁ Targeted messaging to at-risk groups ⦁ EMR screening	**Relationships** ⦁ Building patient and community trust ⦁ Practicing patient-centred, shared care
	**Training and Education** ⦁ Respiratory technician training ⦁ Cultural safety training	

### Sustain and share

Since 2019, the clinic has focused on sustaining care capacity and accessibility. Medical education opportunities are continually offered to enhance the practice of its clinicians, enhance service offerings where available, and continuing to integrate community assets. These actions characterize the current program and its important shift to a strengths-based intervention that identifies and capitalizes on individual and community assets. This starkly contrasts the deficit approach evident in 2012 that had focused on the population problems experienced as exacerbations of complex chronic disease.

### Program outcomes and impacts

**Fig. 3:**
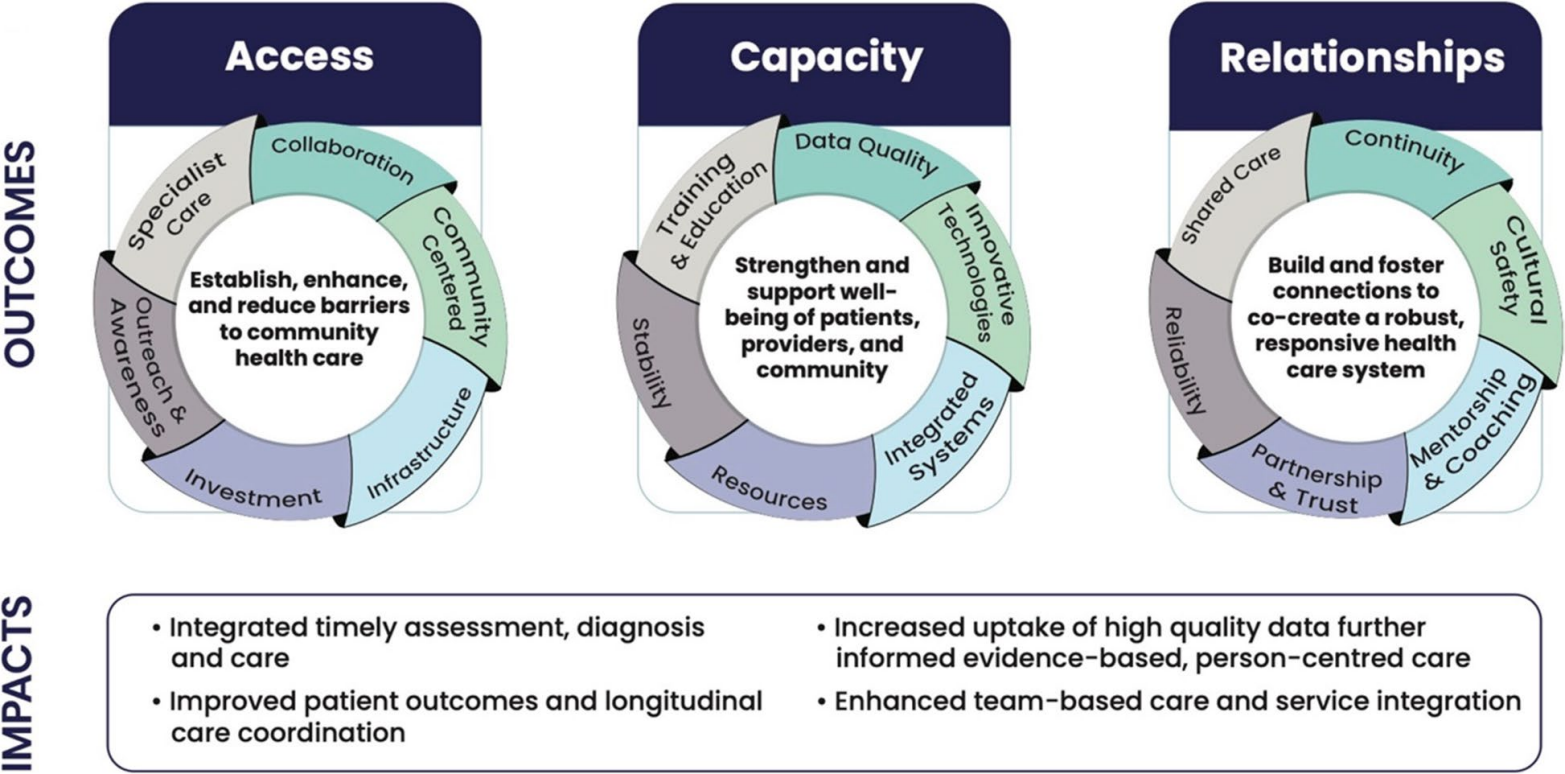
Rural and Northern Community Focused Model of COPD Care (RaNCoM) program outcomes and impacts

Key areas of success identified in RaNCoM were access, capacity building, and relationships (Fig. 3). Access was achieved through establishing, enhancing, and reducing barriers to community healthcare. For instance, the patient population was engaged through strategic and methodical outreach activities such as EMR screening and targeted messaging to at-risk groups, ensuring the community was aware of the services available, and arranging remote community visits on a regular basis. In this sense, program accessibility planning was community-centred. Several interviewees expressed their confidence in the community knowing the clinic existed and was prepared to support high quality COPD care.

For our region, bringing the spirometry to the patient is, I think, part of how this program is different. It’s more patient-oriented, compared to me having to refer you two hours down the road to go get a spirometry test just to tell me, ‘Yes, you qualify for this puffer through Pharmacare.’ (Care Provider 4).

Investment in in-house spirometry testing and specialist care was critical to managing the COPD disease trajectory in the rural community as it reduced patient travel and saved patient's time and money in seeking critical care. In-house spirometry also improved patient access to medication, as the test provides a gold-standard diagnosis that insurance companies require to cover the costs of treatment supports – many of which have a substantial cost that if not covered would not be affordable. As medication is a large component of COPD self-management, access significantly contributes to the next key success of capacity building.

RaNCoM's contract model successfully attracted physicians to the clinic, secured time for them to focus on their patients, and allowed career viability in a rural, northern environment through an Alternative Payment Plan. These resources provided health care stability to the community. Capturing quality data helped to inform program managers of how the program was operating and where it could be improved, ultimately building the capacity of the program itself. Moreover, the successful maintenance of these processes and practices may be attributed to the team-based approach to sharing the workload of the rigorous documentation. Improving self-efficacy included motivational interviewing, training and disease education with patients toward effective self-management and control, to move away from reliance on the physician. Some care provider interviewees observed that patients improved self-management by knowing where and when to go to get medication refills.

[Patients] are [seeking health care support] prior to the exacerbation or needing hospital admission. So, obviously there’s education that has happened from physicians. And phone calls as well, saying, ‘How do I access my medications? I know I’m going to get sick if I don’t have them.’ (Care Provider 5)

Coaching involving personalized COPD Action Plans provided patients with a clearly defined framework to address COPD exacerbations experienced at home through use of emergency flare-up medications and other actions before seeing their most responsible provider in clinic. Self-efficacy was also relevant to clinic staff, represented by the fact that almost all staff interviewees explicitly stated that the team and themselves were “doing their best” in their individual approach to care provision and responding to community needs. Capacity-building successes improved patient outcomes, as evidenced by clinic staff testimonies of decreased COPD exacerbations witnessed in the Emergency Department, increased patient self-management, and implementation of longitudinal care. These efforts were maintained through integrated systems such as shared EMR procedures to enhance patient wellness.

Finally, fostering authentic relationships was foundational to providing reliability and continuity of RaNCoM. Patient and community trust was cultivated through responding to needs indicated from EMR reports and assessment procedures, such as patient and provider experience surveys. Medical education training around cultural humility and safety allowed for an enhanced approach to effective care provision that took account of lived realities and appropriate medical conduct.

So, by us going to community, we’re now servicing you in your community…[and] that’s been huge. It was building trust in community. ‘I might trust you in [Community A] but now you’re in [Community B] – do I trust you in [Community B]?’ So it’s building relationships [through] outreach. (Management Staff 2)

Collaborative networking expanded the outreach and impact of the program through working with local First Nations communities and health authority partners to ensure physician contracts were secured and that community needs were both identified and served. This networking also led to establishing telehealth and remote care services and fostering a new partnership with a hospital in an urban centre to provide efficient spirometry test results. Significant to this theme were patient engagement efforts through the patient recall system and disease management mentorship and coaching, which created dedicated time with physicians and support staff, ultimately fostering patient-provider relationships and an environment of shared care.

### Program challenges and improvements

Program challenges were identified in similar thematic categories to those in program outcomes. That is, the three primary challenges or barriers were related to program access, capacity, or relationship issues. Continuous barriers to program access included transportation challenges due to a lack of means – patients may not own a vehicle, are unable to afford public transport, or there is no transportation to access – which can inhibit a patient coming into the clinic. A lack of pharmacy access on weekends was also identified as impacting medication supports to assist flare-ups and COPD self-management. Capacity challenges included staffing issues like continued staff turnover, limited human resources in a small community, and the need for relevant training to fill those jobs, which not everyone is interested in pursuing.

Relationship issues were the most widely acknowledged challenges by interviewees, particularly between patients and care providers. Patient distrust was recognized as stemming from bad past experiences due to cultural safety issues or the lack of a reliable relationship with previous physicians. Patient lived realities, such as low socio-economic status and work requirements, were additional factors that could inhibit a person from seeking care or missing appointments. Our patient interviewee also indicated the lack of awareness of being involved in a program designated to their disease management. They felt their assigned care provider was limited in their abilities or desire to help, resulting in the patient seeking their own specialist care to meet their needs. While this patient’s experience does not reflect the practice of all clinic staff, it demonstrates a diversity in the care provided. At a broader level, buy-in from decision-making authorities was also seen as a challenge to secure sufficient funding for human resource and care capacity expansion. One interviewee summarized the heart of issue in approaching the complex nature of these challenges:

[To address these challenges], I think we have to look at it from a larger scope: How do you convince a health authority that it’s the right thing to do? How do you convince the physician that it’s the right thing to do? (Management Staff 1)

Program improvements were also identified. First, additional metrics to enhance the measurement of program efficacy were proposed related to treatment (e.g., spirometry status, smoking cessation, intubation, and prescription) as well as understanding the program’s economic impact at a provincial level. Second, continued program monitoring such as ongoing program assessment, ensuring system recalls and data intake procedures were desired to be upheld based on their proven efficacy. Finally, interviewees also mentioned clinic activities underway that aimed to enhance patient outreach, program access, and building community trust while advocating for equity in rural healthcare and gaining buy-in from decision-makers to fund these kinds of initiatives. These improvements reflect the “Sustain and Share” stage of program development, and highlight the team’s commitment to providing effective, longitudinal care to this rural and northern community.

## Discussion

The aim of this qualitative study was to describe the new innovative model of rural COPD service delivery in a rural and northern community in Canada, and investigate stakeholder perceptions of program outcomes and impact. Moore and colleagues’ process evaluation framework was used to guide this work, through which areas of innovation were identified to demonstrate important contextual consideration through a model representation of the current RaNCoM and its synthesis of all key findings.

RaNCoM encapsulates the areas of innovation intrinsic to this program (Fig. 1). The RaNCoM emphasizes at its heart patient and community-centered COPD care. This is supported by the relational characteristics of trust, compassion, care, and commitment which foster patient safety, support desire to seek health support, and establish long-term resources, practices and planning for patient and community wellness. The relational core is the most vital element of this program – it *gives* life to it. In a small community where the boundaries between care provider and patient run thin because of their shared roles as community members, this human factor requires special attention that this program is structured to support. This relational core of RaNCoM is supported by integral resources, which are implemented in accordance with the three program outcomes of relationships, capacity, and access. To sustain the responsive nature of this program, RaNCoM is surrounded by a continuous process that assesses and enhances program resources and outcomes. In this way, the COPD program responds to community needs in an ongoing, longitudinally oriented approach fueled by its relational and collaborative environment.

The ethos of the patient-centered, shared-care model centres the patient within wrap-around services, includes the patient in the care process, and recognizes the value of the patient’s agency over their livelihood. This ethos also frames the care approach to connect all service providers into a team, which mindfully serves patients and the wider community, and actively works to build an “ecosystem of health care” (Management Staff 4). The importance of aa patiente centred care approach to patient management is critical to ensure support of patient preferences, information and education, access to care, emotional support, physical comfort and coordination of care. RaNCoM represents a strengths-based intervention, transformed from one that was formerly deficit-based. Importantly, because it is situated within a rural care context, RaNCoM is both patient-centered and community-centered to be effective. Patient needs are recognized as inextricably nested within and tied to community needs, and work in partnership with community stakeholders towards addressing them creates this responsive process. As such, this responsive process works toward community betterment for all including both patients and care providers.

Ideal implementation of the COPD program requires integral resources to provide optimal patient and community care. These resources are categorized as infrastructure, software, and operational resources. Infrastructure includes an adequate facility with space to conduct program activities, equipment to conduct tests and support operation, medications for disease management, and funds to purchase these items and allow the program to operate. Software includes a shared electronic medical record and quality data. These items ensure care providers have access to consistent and reliable patient information; can securely house this information in a convenient and longitudinal capacity; and can easily pull reports to interpret and learn from what has been collected. Operational resources dictate efficient and effective use of infrastructure and software. These resources include evidence-based procedures and practices to guide professional conduct and patient service, to support a systematic approach to quality assessment and to improve processes through implementing Plan-Do-Study-Act (PDSA) cycles [11]. Workflow architecture ensures shared understanding of care processes that support patients (e.g., referrals). Further, a care network that connects clinic staff, community services, the health authority, and surrounding community health centres, creates a diverse and resilient healthcare ecosystem. Shared values among clinic staff (e.g., compassion, trust, commitment, care) also work to unite patient supports into a team that values one another and works together for the benefit of the patient.

## Conclusion

COPD is a prominent disease across communities in northern communities and one of the leading causes of hospital admission and death in Canada. Successful health interventions are imperative to improving the disease trajectory and supporting patient outcomes. The evaluation of RaNCoM demonstrates that community-based initiatives can make big impacts in the health of rural and remote communities and effectively manage complex, chronic conditions.

## Data Availability

The datasets generated and analysed during the current study are not publically available but may be available from the corresponding author upon reasonable request.
